# Advances and Challenges in Aerobic Granular Sludge Membrane Bioreactors for Treating Sulfamethoxazole in Wastewater

**DOI:** 10.3390/membranes16040139

**Published:** 2026-04-01

**Authors:** Qingyu Zhang, Bingjie Yan, Xinhao Sun, Zhengda Lin, Lu Liu, Haijuan Guo, Fang Ma

**Affiliations:** 1 State Key Laboratory of Urban Water Resource and Environment, School of Environment, Harbin Institute of Technology, Harbin 150090, China; 2 School of Environmental Science, Liaoning University, Shenyang 110031, China

**Keywords:** aerobic granular sludge membrane bioreactor, sulfamethoxazole, antibiotic degradation, extracellular polymeric substances, membrane fouling

## Abstract

Sulfamethoxazole (SMX) is one of the most frequently detected antibiotics in aquatic environments and is difficult to remove by conventional biological treatment because of its persistence, potential toxicity to microbial communities, and associated risk of antibiotic resistance selection. Aerobic granular sludge membrane bioreactors (AGMBRs), which combine the compact and stratified structure of aerobic granular sludge with membrane-based solid–liquid separation, have emerged as a promising platform for SMX-contaminated wastewater treatment because they provide high biomass retention, decoupled sludge retention time (SRT) and hydraulic retention time (HRT), and stable effluent quality. This review systematically summarizes recent advances in AGMBRs for SMX removal, with emphasis on how operating parameters (e.g., dissolved oxygen, hydraulic retention time, organic loading rate, C/N ratio, and sludge retention time) and membrane-related factors (e.g., membrane flux, aeration-induced shear, membrane type, and pore size) affect treatment performance and process stability. The main SMX attenuation pathways in AGMBRs are discussed from three perspectives: sorption and partitioning within granules and extracellular polymeric substances (EPSs), microbial biodegradation and co-metabolism, and membrane retention that prolongs effective contact time and shapes microbial ecology. Particular attention is given to the dual role of EPS and soluble microbial products (SMPs), which contribute to granule stability and SMX tolerance but also accelerate membrane fouling through cake-layer formation, pore blocking, and transmembrane pressure increase. Current challenges include incomplete understanding of transformation products, ARG- and MGE-related risks, long-term fouling–biodegradation interactions, and the lack of pilot-scale validation. Future research should therefore focus on mechanism clarification, integrated control of removal and fouling, energy-efficient operation, and scale-up of AGMBRs for practical antibiotic wastewater treatment.

## 1. Introduction

Safeguarding drinking-water safety from emerging contaminants has become a priority alongside conventional pollution control [1,2,3,4]. Among these contaminants, antibiotics persist through standard treatment and are frequently detected in receiving waters [5,6,7,8,9,10,11]. A major recent concern is the widespread occurrence of antibiotics, such as sulfamethoxazole (SMX), in wastewater effluents [6], surface water [7], and even groundwater [8]. SMX is frequently detected at roughly 0.1–2.5 µg L^−1^ in wastewater effluents, typically 10 ng L^−1^ (median ≈ 50 ng L^−1^) but occasionally up to 4–6 µg L^−1^ in surface waters, and about 12.7–258 ng L^−1^ in groundwater, reflecting incomplete removal by standard treatment processes [9]. SMX is a high-frequency sulfonamide in municipal/hospital and livestock effluents, is relatively recalcitrant, and exerts selection pressure for sul-type ARGs [5,9,10,11]. These features make SMX a stringent and representative target for evaluating antibiotic removal technologies.

Aerobic granular sludge (AGS) technology has emerged as a promising biological platform to meet these challenges [12]. AGS is characterized by its dense, compact structure, smooth surface, and well-defined pores [13], with granule sizes typically ranging from 0.3 to 4.5 mm. These properties enable efficient biomass retention, effective separation from treated water, and settleability superior to that of conventional floccular sludge [14,15]. Compared to conventional floccular sludge, AGS allows higher biomass concentrations, enhanced removal of nitrogen, phosphorus, and organic pollutants in a single tank, and improved resistance to organic and hydraulic shock loads [14,16,17,18]. Moreover, its large surface area, abundant pores, and EPS secretion promote adsorption of heavy metals and toxic substances, facilitating their removal from wastewater [19]. The unique anaerobic and aerobic microenvironments within AGS support simultaneous nitrification and denitrification, which is particularly beneficial for high-nitrogen wastewaters [20,21].

Building on these advantages, aerobic granular sludge membrane bioreactors (AGMBRs) integrate AGS with membrane-based solid–liquid separation to further intensify treatment for SMX-contaminated wastewater. In AGMBRs, the membrane barrier stabilizes effluent quality and enables flexible control of sludge retention time relative to hydraulic retention time, while the granular architecture provides stratified micro-niches and strong stress tolerance that can benefit antibiotic attenuation. At the same time, membrane integration introduces new constraints—most notably membrane fouling—making the interactions among SMX exposure, granule properties, and extracellular polymeric substances (EPSs) central to both pollutant removal and operational stability.

**Figure 1:**
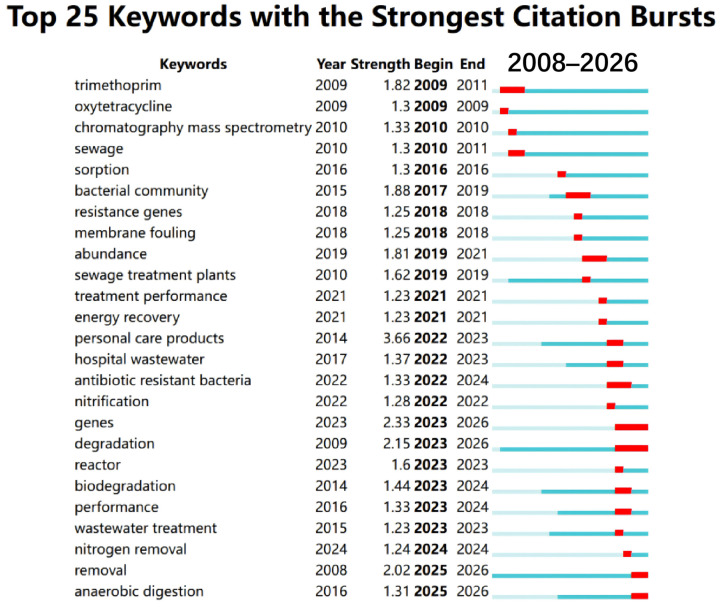
Top 25 keywords with the strongest citation bursts (CiteSpace) in the MBR–antibiotics–sludge literature based on Web of Science records.

**Figure 2:**
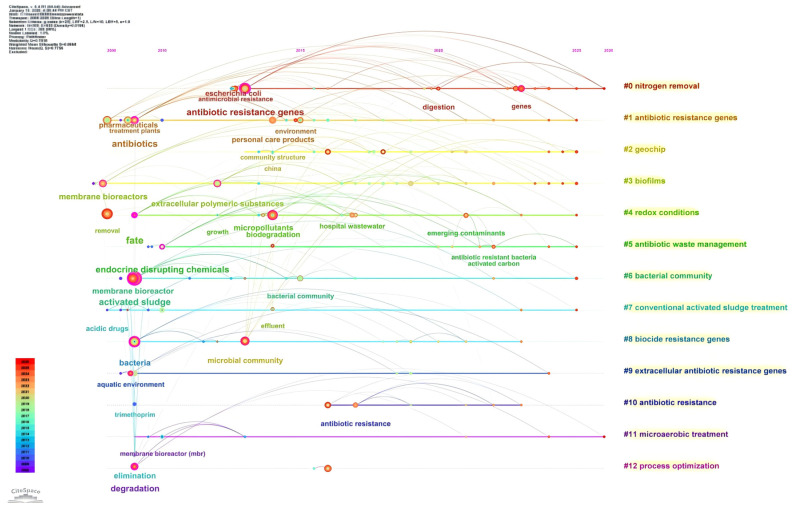
Timeline view of keyword clusters (CiteSpace) showing the temporal evolution of major research themes in the MBR–antibiotics–sludge literature based on Web of Science records.

AGS forms a unique biofilm structure via aerobic self-aggregation, yielding high biomass retention and a diverse microbial community. This enables AGS systems (and, by extension, AGMBRs that employ granular sludge as the biological core) to achieve higher organic and nutrient removal than traditional activated sludge, as well as effective biodegradation of refractory compounds, including antibiotics [14,22,23]. To contextualize the evolving research focus on antibiotics in membrane bioreactor (MBR) sludge systems, we conducted a bibliometric analysis using CiteSpace based on a Web of Science Core Collection topic search (TS = “membrane bioreactor” AND “antibiotic” AND “sludge”), which returned 107 records. The full records (with cited references) were exported from Web of Science and imported into CiteSpace, where we set the node type to “Keyword” and performed keyword co-occurrence analysis, burst detection, and timeline clustering. Figure 1 (Top 25 keywords with the strongest citation bursts) is generated directly from CiteSpace’s burst-detection function, which identifies keywords that experience a rapid increase in citations/occurrence over specific time windows, thereby highlighting stage-specific research fronts. Figure 2 (timeline visualization of keyword clusters) is produced by CiteSpace’s keyword clustering and timeline view, which organizes major thematic clusters and displays their temporal evolution and interconnections. Together, these two figures reveal a clear shift in the field from early emphasis on analytical detection and the fate of representative antibiotics (e.g., trimethoprim; chromatography–mass spectrometry) toward process-limiting mechanisms (sorption, membrane fouling) and, more recently, to microbial ecology and resistance risks (bacterial community, ARG/ARB, hospital wastewater), alongside an emerging systems-level agenda that couples antibiotic-risk control with core treatment objectives such as nitrogen removal, anaerobic digestion, and energy recovery [24]. Despite rapid advances, the resistance and adaptability of granular sludge to antibiotics like SMX—and their implications for stable operation in membrane-coupled systems—remain underexplored [25]. Antibiotic exposure can alter microbial community structure, reduce treatment efficiency, and select for resistant bacteria within granular sludge systems [26]. Thus, clarifying how antibiotic stress reshapes granule integrity, EPS production, and community function is essential for the sustainable application and scale-up of AGMBRs.

In this context, this review provides a focused and updated synthesis of recent advances and remaining challenges in AGMBRs for treating SMX-contaminated wastewater. Unlike previous reviews that mainly address aerobic granular sludge, conventional membrane bioreactors, or antibiotic removal in a broader sense, this work specifically emphasizes the membrane-coupled granular system as an integrated platform in which pollutant attenuation, granule stability, EPS/SMP dynamics, and membrane fouling are tightly interconnected. Particular attention is given to how SMX stress reshapes the biological and physicochemical behavior of AGMBRs, including microbial adaptation, granule integrity, removal pathways, and filtration stability. Accordingly, the following sections first summarize the biological foundation of AGMBRs through granular sludge formation and stability, then examine the impacts of SMX on granule function and system performance, analyze the adaptability and degradation capacity of granular sludge under antibiotic stress, discuss the key operational and water-quality factors governing SMX removal and membrane stability, and finally outline future research priorities for mechanism clarification, fouling control, ARG-risk management, and scale-up. By centering on the coupling relationships among SMX removal, granular sludge responses, and membrane performance, this review aims to provide a clearer framework for understanding AGMBR behavior and for guiding the design and optimization of practical antibiotic wastewater treatment systems.

## 2. Formation Mechanisms of Granular Sludge in AGMBRs

**Figure 3:**
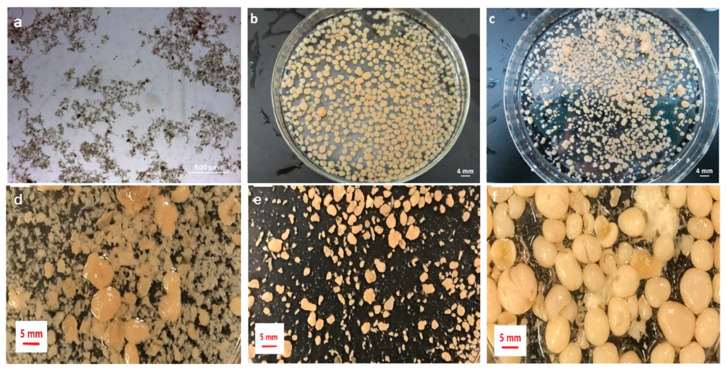
Morphology of the sludge. (**a**) seed sludge; (**b**) granules in R_1_ at stabilization phase; (**c**) granules in R_2_ at stabilization phase [30]; (**d**) AGS on Day 61 in AN/O/AX/O_SBR; (**e**) AGS on Day 109 in AN/O_SBR; (**f**) AGS on Day 61 in O_SBR [31].

**Figure 4:**
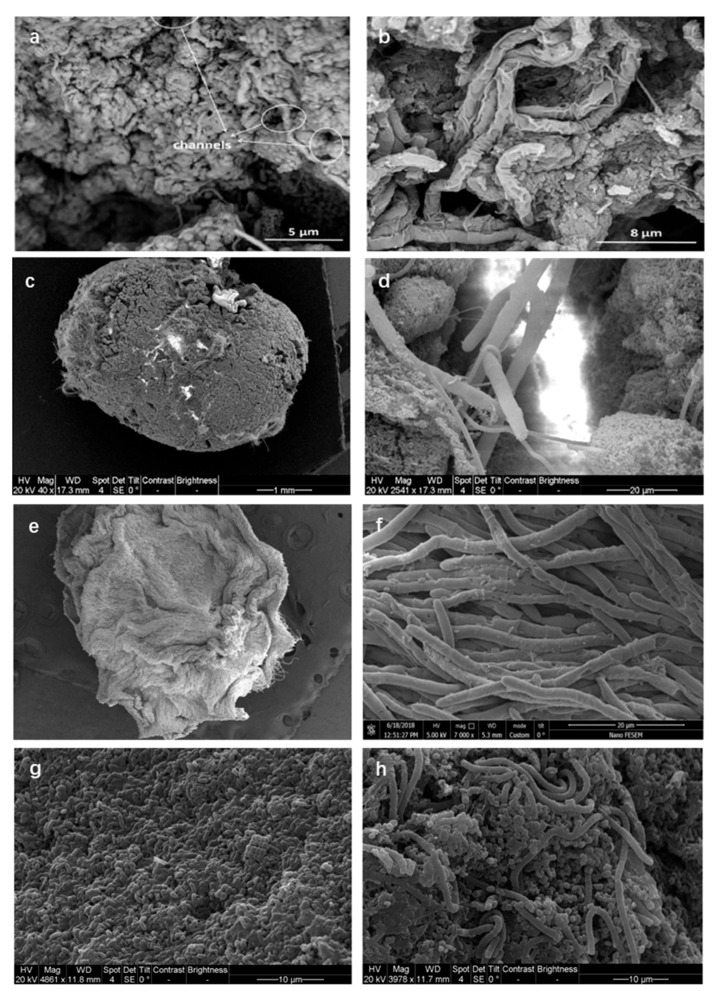
SEM image of AGS: (**a**) the outer surface of granules; (**b**) the internal structure of granules [30]; (**c**) mature granules in AN/O/AX/O_SBR; (**d**) surface amplification of mature particles in AN/O/AX/O_SBR [31]; (**e**) AGS without nanoparticles full view; (**f**) AGS nanoparticle-free magnification [32]; (**g**) granule at 5000 magnification; (**h**) granule at 4000 magnification [33].

AGS differs morphologically from conventional activated sludge, typically forming light-yellow to orange spherical/ellipsoidal granules with relatively smooth surfaces and diameters from a few millimeters to several centimeters; size/shape uniformity supports settling and separation, while smooth surfaces facilitate oxygen and substrate transfer essential for metabolism and removal [27,28,29]. Across SBR configurations, stable AGS routinely forms (Figure 3a–f), as demonstrated in two identical cylindrical reactors and in alternative set-ups [30,31]. Microstructures span filament-free, pore-channeled granules to feather-like, filament-rich surfaces with core fungal networks that entrap nematodes, as well as EPS-reinforced compact architectures and rod-dominated surfaces where filaments serve as connectors (Figure 4a–h) [30,31,32,33]. AGS is characterized by a stratified internal structure, consisting of an outer aerobic layer enriched in nitrifiers and nitrite-oxidizers, an intermediate anoxic zone favorable for denitrification, and inner anaerobic regions inhabited by organisms such as sulfate-reducers, thereby enabling simultaneous nitrification, denitrification, and organic matter removal [34,35,36,37,38].

In the context of AGMBRs, these formation principles remain the biological basis for process intensification and stable effluent quality. However, membrane coupling changes the operational constraints that ultimately determine whether granulation is sustainable: while classical AGS relies strongly on hydraulic selection through settling and washout, AGMBRs can retain slower-settling biomass via membrane separation, potentially weakening physical selection pressure and shifting the balance toward shear-driven aggregation, EPS-mediated cohesion, and substrate/oxygen gradients within granules. Therefore, understanding AGS formation mechanisms is essential not only for generating robust granules but also for controlling granule integrity, EPS/SMP release, and fouling propensity—all of which directly affect AGMBR stability and SMX treatment performance.

### 2.1. Bacterial Self-Aggregation Theory

The bacterial self-aggregation theory emphasizes the spontaneous aggregation phenomenon of microorganisms under appropriate conditions. Under the influence of various factors such as high shear force and pH, microorganisms form tight aggregates through physical and chemical interactions with each other [39]. The formation of AGS is a gradual process involving the densification and aggregation of seed sludge, ultimately resulting in a stable three-dimensional structure [31,40]. In order to enhance the nitrogen and phosphorus removal capabilities of the low-loading AGS-SBR system, tuning the C/N ratio modulates AGS morphology and EPS, enabling simultaneous removal of organics, nitrogen, and phosphorus under low loading [41]. This experiment validates the effectiveness of the bacterial self-aggregation theory in practical applications. The morphology of the inoculated seed sludge and the acclimated biomass in the SBR is shown in Figure 3a–c [30]. These images capture the progression towards a stable AGS structure. Figure 3d–f further document the mature granules obtained after successful cultivation [31]. Across pilot-scale GSBRs, influent wastewater strength—and the resulting organic and sludge loading—strongly governs both the granulation time and the final granule size [31]. Using multicolor fluorescence in situ hybridization technology, the internal structures of freshly inoculated sludge and mature granular sludge were examined. Mingyue Geng et al. cultured AGS in SBR. As shown in Figure 4a,b, no obvious filaments were observed on the surface of the mature granules during the stable stage, and bacteria-dominated granules were obtained. Some hyphae were observed to remain inside the particles, resulting in the formation of pores and channels [30]. As shown in Figure 4c,d, Roya et al. cultivated AGS in an AN/O/AX/O-SBR system and observed feather-like growth of filamentous bacteria on the surface of the aerobic granules. Extensive fungal hyphae were rooted in the core of these granules, forming a network that trapped large nematodes. An enlarged image of the cracks on the particle surfaces revealed the interwoven network of fungi and filamentous bacteria. The particle surfaces were predominantly occupied by tiny rod-shaped bacteria [31].

The results of morphological analysis of AGS by Hasti et al. were shown in Figure 4e,f. AGS is surrounded by strong EPS bundles and a high number of filamentous bacteria. Filamentous bacteria are the dominant bacteria on the surface of the particles, which create a very compact structure for AGS. EPS and filaments have a protective effect on AGS [32]. The AGS cultivated by Iorhemen Oliver et al. was displayed in Figure 4g,h. Examine the surface morphology, where high-concentration bacteria prevail, predominantly rod-shaped ones, with some filamentous bacteria also present. These filamentous bacteria serve as connectors, linking other bacterial species within the AGS matrix [33]. It was found that microbial self-aggregation is the initial step in the formation of granular sludge. The aggregated microorganisms secrete EPS at attachment points, proliferate to promote sludge growth, and ultimately form granular sludge [42].

For AGMBRs, self-aggregation is particularly relevant due to the unique biomass retention and hydrodynamic environment of membrane-coupled systems: membrane retention can maintain high biomass concentrations and prolong contact among cells, potentially accelerating early aggregation even when settling-based selection is relaxed. Unlike conventional AGS systems that rely on hydraulic washout for screening, AGMBRs retain all suspended biomass via membrane filtration, so loose aggregates formed in the early stage cannot be eliminated and will continuously release colloidal biopolymers. At the same time, excessive accumulation of loosely bound aggregates may increase soluble and colloidal biopolymers, which directly increase the fouling load on the membrane surface and deteriorate filtration stability, linking early-stage aggregation dynamics to later membrane fouling risk.

### 2.2. Cell Surface Hydrophobicity Hypothesis

The cell surface hydrophobicity hypothesis suggests that the surface hydrophobicity of sludge varies due to differences in culturing methods or influent substrates [43]. Cell surface hydrophobicity is related to surface Gibbs energy and intercellular affinity, promoting bacterial aggregation into clusters and detachment from the aqueous phase to cultivate AGS. When glucose or acetate serves as the sole carbon source, surface hydrophobicity increases markedly relative to suspended sludge, favoring aggregation and phase detachment [44,45]. This result is consistent with the cellular surface hydrophobicity hypothesis.

In AGMBRs, shifts in cell surface hydrophobicity not only influence granule formation but can also affect granule–membrane and foulant–membrane interactions, the hydrophobic attraction between biomass and membrane surface will accelerate the deposition of foulants, while hydrophilic surfaces help mitigate irreversible fouling, thereby coupling granulation propensity with filtration behavior and cleaning reversibility. This physical interaction mechanism is unique to membrane-coupled systems and does not exist in conventional AGS reactors.

### 2.3. EPS Promotion Hypothesis

EPSs are high-molecular-weight substances secreted by microorganisms, composed of proteins (PN), polysaccharides (PS), and others. EPSs bind to microorganisms through ionic bonds, coordination bonds, and other means, facilitating the adhesion of bacteria-to-bacteria and bacteria-to-solids, forming aggregation nuclei [46]. EPS plays a crucial role in the development of AGS, capable of altering the surface charge and hydrophobicity of cells, thereby promoting microbial aggregation and the formation of granular sludge [47]. Experiments have found that mature granular sludge has PN as its core and β-polysaccharides as its skeleton, with increased PS content enhancing the stability of granular sludge. CLSM shows that mature granules commonly feature a PN-rich core with a β-polysaccharide skeleton, and higher PS content enhances structural stability [48]. This result supports the extracellular polymeric substance (EPS) promotion hypothesis.

For AGMBRs treating SMX, EPS is a “double-edged sword”: it strengthens granule cohesion and creates diffusion–reaction microenvironments beneficial for biodegradation, yet EPS and related soluble microbial products can also act as key membrane-fouling precursors. In conventional AGS systems, excess EPS only affects sludge settleability; in AGMBRs, however, EPS/SMP will be intercepted by the membrane and form a dense cake layer, causing a sharp rise in transmembrane pressure. Consequently, EPS-mediated granulation should be discussed alongside strategies that manage EPS/SMP release to maintain stable filtration.

### 2.4. Selective Pressure-Driven Hypothesis

The selective pressure-driven hypothesis proposes that, during the cultivation of granular sludge, conditions such as settlement time are controlled to screen out sludge with poor settlement performance, thereby retaining active sludge with better settlement performance to complete granulation. Only the granular sludge with a fast settlement rate can be retained within the reactor, while those with poor settlement performance will be washed out of the reactor [49,50]. This is a purely physical screening process that does not involve microbial responses to the environment. The magnitude of selective pressure determines the granulation rate of aerobic sludge. The increase in selective pressure can gradually enhance the stability of granular sludge. In SBR, sludge granulation was achieved by controlling the settlement time [51]. Shortening the settlement time helps to wash out floc sludge with poor settlement performance, creating a relatively strong selective pressure, which promotes the formation of AGS [52,53].

In AGMBRs, membrane-based separation can partially replace settling-driven washout, meaning that classical “short settling time” selection may be weakened. This is the most critical difference between AGMBRs and conventional AGS in the granulation mechanism. As a result, selective pressure in AGMBRs may shift toward hydrodynamic shear, feast–famine regimes, and operational control of biomass inventory, which together determine whether stable granules dominate or whether mixed flocs/fragmented granules accumulate—conditions that can markedly influence fouling development and SMX removal stability.

### 2.5. Complexity of the Formation Process

**Figure 5:**
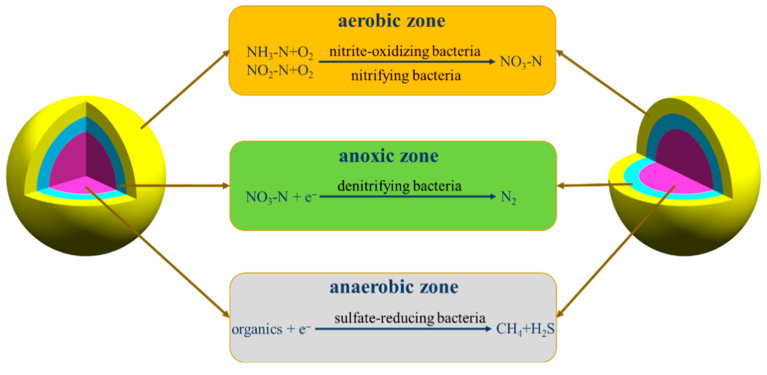
Internal stratification diagram of AGS (the stratification is physically governed by oxygen/substrate diffusion resistance across the granular porous structure, forming aerobic, anoxic and anaerobic physical micro-niches from the surface to the core).

**Figure 6:**
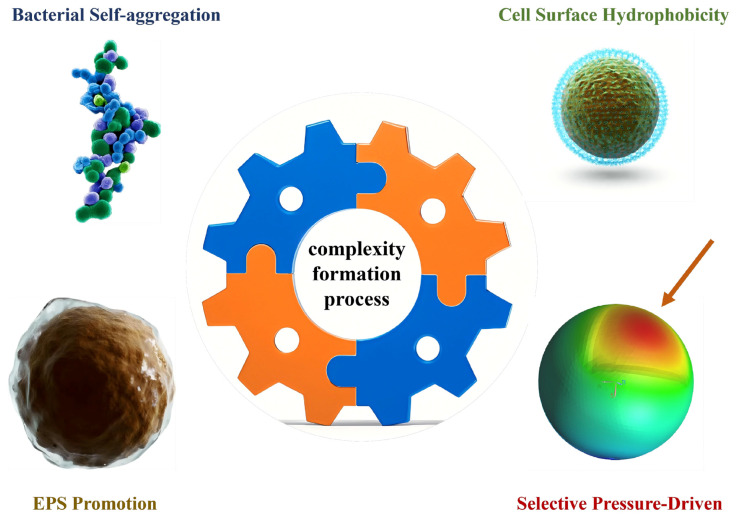
Physical–biological coupled formation mechanism of AGS (physical drivers include hydraulic shear, surface hydrophobicity, ionic bridging and physical selective pressure, which synergize with biological processes to drive granulation).

The internal stratification of AGS is shown in Figure 5. Physically, this stratified architecture is driven by the gradient diffusion of oxygen, substrates and metabolites across the granular porous structure, which creates distinct mass-transfer resistance layers and physical micro-niches from the exterior to the interior of granules. The dense outer layer imposes high physical diffusion resistance to form the aerobic zone; the middle layer with moderate oxygen flux constitutes the anoxic zone; and the inner core with low permeability becomes the anaerobic zone due to complete oxygen depletion. The formation of AGS is an extremely complex process, involving the intertwined influences of physical, chemical, and biological factors [53]. During this process, physical factors such as hydraulic shear force and diffusive forces play crucial roles by facilitating the physical movement of bacteria, thereby laying a solid foundation for the initial formation of granular sludge [54,55]. Quantifying hydraulic shear beyond apparent gas velocity indicates total shear rates of 0.56–2.31 × 10^5^ s^−1^ in high-aspect-ratio reactors—conditions that promote aggregation and enhance granular stability [54]. The results showed that strong hydraulic shear stress promoted microbial aggregation and was beneficial to the structural stability of granular sludge. The sedimentation and washing-out process also made a significant contribution to the formation of granular sludge. Chemical forces like ionic bonds and coordination bonds tightly bind EPS to microorganisms, constructing a stable granular structure [56]. Notably, metal ions such as Ca^2+^ and Mg^2+^ also play significant roles in this process by stimulating the secretion of EPS, thereby further optimizing the properties of the sludge [57]. The physical–biological coupled hypothesis mechanism of AGS formation was shown in Figure 6. From the physical perspective, four key physical forces dominate the granulation process: (1) hydraulic shear force provides physical shaping and compaction for granules; (2) cell surface hydrophobicity induces physical phase separation and cellular aggregation; (3) ionic bridging (e.g., Ca^2+^/Mg^2+^) acts as the physical binding force; (4) settling-based physical selective pressure screens biomass with excellent settleability. These physical processes interact with biological EPS secretion and microbial self-aggregation to promote stable granule formation.

For AGMBRs, this multifactor coupling is further complicated by filtration-induced stresses (e.g., concentration of colloids/biopolymers near the membrane, recycle shear, and aeration scouring), which can feed back to granule stability. Conventional AGS formation is only affected by hydraulic and biological factors, while AGMBRs add membrane filtration as a new regulatory dimension, forming a closed-loop interaction of “granulation → EPS release → membrane fouling → shear change → granule structure adjustment”. Therefore, when applying AGMBRs to SMX-contaminated wastewater, granulation strategies should be evaluated together with membrane-operation constraints to achieve a balanced outcome: robust granules, controlled EPS/SMP release, and sustainable filtration performance.

## 3. Impacts of Antibiotics Such as Sulfamethoxazole on Granular Sludge in AGMBRs

In future water treatment, AGMBRs are increasingly viewed as a promising platform for antibiotic-contaminated wastewater because they combine the compact, shock-resistant characteristics of granular sludge with membrane-based solid–liquid separation, enabling stable effluent quality and high biomass retention. However, with the widespread use of antibiotics such as SMX and their long-term residence in aquatic environments, AGMBR operation faces compounded challenges that extend beyond those observed in stand-alone AGS systems [58,59]. In addition to directly inhibiting microbial activity and destabilizing granule structure, SMX-induced shifts in extracellular polymeric substances (EPSs), soluble microbial products, and particle size distribution can accelerate membrane fouling and undermine filtration stability. Accordingly, this section discusses how antibiotics like SMX affect granular sludge function in AGMBRs, focusing on inhibition of microbial activity, impacts on structural stability, and downstream consequences for settling/particle integrity, effluent quality, and membrane-related performance.

### 3.1. Inhibitory Effects of Antibiotics on Microbial Activity in Granular Sludge

The presence of antibiotics significantly affects sludge microbial activity. SMX, as a broad-spectrum sulfonamide antibiotic, exhibits characteristics such as low adsorption, difficulty in degradation, and ease of accumulation, which can inhibit the growth and metabolism of microorganisms in sludge. At trace SMX (5 μg/L), AGS can outperform SMX-free controls in conventional pollutant removal, indicating a potential hormetic response [60]. Over a continuous 240-day test, elevated SMX at 1.0 and 5.0 mg/L significantly inhibited microbial growth and nitrification, weakened sludge settleability, suppressed EPS secretion, and promoted the proliferation of ARGs, with Thiothrix becoming the dominant genus in AGS [61]. In another study, the relative abundance of Flavobacterium gradually decreased with increasing SMX concentration [62].

SMX can significantly inhibit the growth of certain key microorganisms in granular sludge, such as nitrifying and denitrifying bacteria, which play crucial roles in the nitrogen cycle [63,64]. Furthermore, SMX may further suppress microbial activity by damaging microbial cell membranes or interfering with the replication of genetic material. In AGMBRs, membrane retention can increase the effective residence time of both biomass and pollutants, which may partially buffer short-term shocks but can also prolong exposure and magnify chronic inhibition, making community stability and functional redundancy especially important under sustained SMX loading.

### 3.2. Influence of Antibiotics on Granule Structural Stability and Its Implications for Filtration

The impact of antibiotics on sludge structural stability cannot be overlooked. The presence of SMX leads to the disintegration of sludge flocs and cell rupture, resulting in an increase in the total amount of EPS and its main components, proteins and polysaccharides, to form a protective barrier [63,64,65,66]. These changes not only affect sludge structural stability but also reduce its capacity to treat wastewater [67]. The introduction of antibiotics can disrupt this balance, leading to the disintegration of the granular structure [47,68,69]. EPSs are a crucial component of granular sludge, playing a key role in sludge flocculation, settlement, and biofilm formation [70].

In addition to hydraulic and permeability penalties, membrane-associated foulant layers may represent localized “hotspots” for antibiotic exposure and resistance selection. Studies on MBRs exposed to antibiotics indicate that a substantial fraction of ARGs can accumulate in membrane foulants, and antibiotic additions can increase ARG abundance in both sludge and the fouling layer, highlighting that fouling is not only an operational issue but also a potential AMR-risk compartment [71,72]. Consistent with this risk perspective, multi-omics evidence from an SMX-stressed continuous-flow AGS–MBR shows enrichment of sul-type ARGs and signals linked to horizontal gene transfer under SMX exposure, implying that SMX retention/partitioning within EPS and membrane foulants may contribute to local selection pressure in AGMBRs. Therefore, SMX removal and fouling control should be jointly evaluated with resistome/mobilome endpoints when assessing environmental safety of AGMBR operation.

From an AGMBR perspective, these SMX-driven structural responses have an additional operational consequence: granule breakage and elevated EPS production can increase the abundance of fine particles and colloids, thereby increasing the load of potential foulants delivered to the membrane surface. Thus, the same protective response that helps microorganisms tolerate SMX (e.g., higher EPS) may simultaneously increase filtration resistance and destabilize membrane operation. This coupling between granule integrity–EPS dynamics–membrane fouling propensity is a defining feature that distinguishes SMX impacts in AGMBRs from those in conventional AGS reactors.

### 3.3. Effects on Particle Integrity, Effluent Quality, and Overall AGMBR Stability

The presence of antibiotics has a significant negative impact on sludge settling performance and effluent water quality. The residue of SMX leads to a decrease in the sludge volume index (SVI), smaller sludge particles, and worsened settling performance [73]. Specifically, the total organic carbon (TOC) in the effluent rapidly increases, and the pollutant removal rate decreases [72]. Furthermore, antibiotics may also affect the metabolic pathways of microorganisms in sludge, producing toxic intermediates, further threatening the safety of effluent water quality [74]. The dispersion and fragmentation of sludge particles can lead to a reduction in settlement velocity and an increase in sludge volume, thereby increasing the difficulty and cost of subsequent treatment [75]. Especially for pollutants with structures similar to antibiotics, the removal effectiveness of granular sludge may be even more significantly affected [76].

While membrane separation in AGMBRs reduces reliance on gravity settling for solid–liquid separation, SMX-driven particle fragmentation and increased dissolved/colloidal organics can still compromise process stability by elevating the foulant burden and potentially deteriorating permeate quality (e.g., higher TOC) [77]. Therefore, antibiotic impacts in AGMBRs should be understood as a combined effect on biological conversion capacity (microbial inhibition and pathway perturbation) and physical filtration stability (changes in particle size distribution and EPS-related foulants).

Overall, the impact of antibiotics such as sulfamethoxazole on granular sludge is multifaceted, including inhibiting microbial activity, affecting structural stability, and reducing particle integrity and effluent water quality, with additional consequences for membrane-related stability in AGMBRs. In future promotion and application of AGMBRs for antibiotic-contaminated wastewater, antibiotic stress must be explicitly considered, and targeted strategies should be developed to mitigate both biological inhibition and filtration instability. For example, by optimizing sludge cultivation conditions [78], screening antibiotic-resistant microbial strains [79], and developing efficient antibiotic removal technologies [80], the tolerance and treatment efficiency of granular sludge towards antibiotics can be improved, helping to ensure safe and stable effluent quality under long-term SMX exposure.

## 4. Adaptability and Degradation Capacity of Granular Sludge in AGMBRs Under Antibiotic Stress

AGMBRs inherit the intrinsic stress tolerance of AGS while adding membrane-based solid–liquid separation that stabilizes effluent quality and enables high biomass retention. Under antibiotic pressure (e.g., SMX), the adaptability and degradation capacity of granular sludge in AGMBRs are governed not only by microbial selection and metabolic versatility but also by membrane-coupled operational constraints (e.g., biomass inventory, soluble/colloidal biopolymers, and filtration stability). This section summarizes how microbial communities adapt under antibiotics, how key microorganisms contribute to antibiotic degradation within granules, and which engineering strategies can further enhance SMX attenuation in AGMBRs while maintaining operational stability.

### 4.1. Adaptive Changes in Microbial Community Structure Under Antibiotics

Granular sludge exhibits pronounced and directed shifts in microbial community composition under antibiotic pressure (e.g., SMX), with adjustments in taxa, abundance, and functional profiles that reflect selection for tolerant and/or degrading populations [81]. Through microbial self-evolution and ecological selection, communities gradually adapt to antibiotic exposure and form tolerant consortia [82]. In such environments, susceptible microorganisms are suppressed, whereas taxa with tolerance or catabolic potential tend to dominate; this community turnover helps maintain the granular framework and supports pollutant removal under antibiotic stress [64,68].

At the genetic level, communities adapt by rewiring metabolism and acquiring resistance via horizontal gene transfer and recombination, facilitating a shift from susceptible to tolerant dominants [83,84]. Correspondingly, dominant species may transition toward more antibiotic-tolerant lineages, which stabilizes treatment performance and sustains system capacity to a practical extent in antibiotic-containing wastewaters [85,86]. In AGMBRs, membrane retention can further intensify these dynamics by prolonging biomass residence time and increasing the likelihood that slow-growing but tolerant or specialized degraders persist, thereby reshaping community succession pathways relative to conventional washout-controlled systems. It should also be noted that operational and environmental conditions (e.g., temperature, pH, dissolved oxygen) modulate these antibiotic-driven dynamics; their individual impacts are analyzed in Section 6.

### 4.2. Mechanisms of Key Microorganisms Degrading Antibiotics Within Granular Sludge

Multiple microbial lineages within granular sludge contribute to antibiotic attenuation via complementary metabolic routes. Broadly, two routes prevail: biotransformation, in which enzymes directly modify or cleave antibiotic molecules, and cometabolism, wherein antibiotic transformation is facilitated during the metabolism of added co-substrates [87,88,89,90]. Specific enzyme systems recognize characteristic bonds and convert target antibiotics into more labile intermediates or smaller molecules; for example, β-lactamases hydrolyze the β-lactam ring of penicillin-type antibiotics and deactivate them [91]. Cometabolic pathways further enhance removal by supplying electron acceptors/donors and co-substrates, or by upregulating auxiliary enzyme sets that broaden catalytic scope [92,93]. During tetracycline removal from swine wastewater, AGS-enriched enzymes are typically associated with aromatic-compound biodegradation, suggesting their participation in TC transformation within the granules [94].

The granular architecture itself aids antibiotic processing: biofilms enlarge contact interfaces between biomass and dissolved antibiotics, while the EPS matrix sorbs and localizes antibiotic molecules, increasing their residence time near active cells and enzyme pools [95,96]. These physical–chemical microenvironments, coupled with metabolic versatility, jointly underpin the observed antibiotic degradation in granular sludge. In AGMBRs, the membrane barrier can further enhance the effective “exposure time” of antibiotics to these microenvironments by retaining particulate/colloidal fractions and stabilizing mixed liquor conditions; however, this benefit must be balanced against the risk that elevated EPS/biopolymers and fine particles—often promoted under antibiotic stress—increase fouling propensity, potentially constraining the operational window for sustained biodegradation.

### 4.3. Strategies to Enhance Antibiotic Degradation Efficiency in AGMBRs

Process optimization. Tuning hydraulic retention time, aeration intensity, and nutrient ratios can selectively enrich dominant degraders, increase active biomass, and improve antibiotic removal [97]. In AGMBRs, such optimization should also account for membrane operation (e.g., maintaining stable biomass characteristics that minimize excessive soluble/colloidal foulants) to avoid trading improved biodegradation for accelerated filtration deterioration.

Cometabolic stimulation. Introducing readily biodegradable co-substrates promotes cometabolic pathways and boosts transformation rates of target antibiotics without altering core reactor configuration [98,99]. For AGMBRs, careful selection and dosing of co-substrates are important to prevent overproduction of SMP/EPS that can aggravate fouling, while still supporting cometabolic transformation.

Microbial and genetic interventions. Targeted manipulation of community structure and genetic potential can enhance catabolic capacity and tolerance. Approaches include enriching or engineering strains with higher antibiotic-degradation capabilities, and steering community composition toward functional guilds that sustain antibiotic removal under stress [100,101]. As a practical example, montmorillonite addition under elevated SMX mitigated ARG generation, enriched Thauera (7.84%), and linked ARG levels to potential carriers such as Paenarthrobacter and Caldilineaceae [102]. Such interventions may be particularly valuable in AGMBRs where long biomass retention can help maintain engineered or enriched functional consortia.

Hybridization with complementary processes. Coupling granular sludge treatment with advanced oxidation or physical–chemical steps can pre-activate or partition antibiotic molecules [103], rendering them more amenable to subsequent biological transformation within granules and thus improving overall antibiotic removal [104,105]. In an AGMBR framework, hybridization can be designed either upstream (to reduce biotoxicity and improve biodegradability) or downstream (to polish residual SMX and transformation products), while the membrane unit ensures consistent solid–liquid separation and supports stable process integration.

Overall, compared with conventional activated sludge, granular sludge-based systems generally maintain higher resilience to antibiotics owing to compact architecture, stratified micro-niches, and a dense EPS matrix. Within AGMBRs, these biological advantages can be further leveraged by membrane-enabled biomass retention, but the benefits are inseparable from membrane-related constraints. By combining process optimization, cometabolic stimulation, community/strain engineering, and judicious hybridization with complementary unit processes, the antibiotic-degradation performance of granular sludge can be strengthened within practical treatment windows while maintaining long-term AGMBR stability.

## 5. Key Factors Influencing Antibiotic Removal Efficiency in AGMBRs

AGMBRs, by integrating granular sludge with membrane-based solid–liquid separation, offer a promising route for treating wastewater containing antibiotics such as sulfamethoxazole. Compared with stand-alone AGS, the performance envelope of AGMBRs is shaped by a tighter coupling between biological conversion (granule integrity, microbial activity, EPS-mediated partitioning/biodegradation) and filtration stability (membrane flux/TMP behavior, fouling propensity). Therefore, the efficiency of antibiotic removal in AGMBRs is profoundly influenced by operational conditions that simultaneously regulate granulation, microbial function, and the generation/transport of foulant precursors.

### 5.1. Operational Conditions

#### 5.1.1. Dissolved Oxygen Concentration

Dissolved oxygen (DO) is one of the key factors affecting the formation and stability of granular sludge. Appropriate DO concentrations help maintain microbial activity within the sludge and promote the formation and stability of granules. Under low DO conditions, it is difficult to successfully initiate a granular sludge system, and granules can only be clearly observed when DO reaches a certain level. Excessively low DO concentrations can lead to gas production within the granules, combined with the proliferation of filamentous bacteria under low DO conditions, which can easily cause internal rupture of granules [106]. Studies have shown that controlling the DO concentration at 50% of its saturation concentration can maintain the stability of the granular structure. In a stable granular sludge system, due to the structural characteristics of the granules, when DO is low, oxygen transfer within the granules is difficult, and anaerobic conditions can easily occur internally, leading to excessive growth of filamentous bacteria and disintegration or flocculation of granular sludge [107]. Additionally, DO has a significant impact on the nitrogen and phosphorus removal efficiency of granular sludge systems, with higher DO levels favoring improved removal rates of carbon, nitrogen, and phosphorus [106]. Raising DO by increasing aeration stabilizes granules by favoring non-filamentous competitors and improving oxygen penetration [108].

During antibiotic degradation, higher DO concentrations may enhance microbial metabolic activity, thereby increasing degradation efficiency. However, excessively high DO concentrations can lead to looser sludge structures, affecting the settlement performance and mechanical integrity of granules. Conversely, low DO conditions may limit microbial respiration, reducing the degradation rate of antibiotics. In AGMBRs, DO additionally interacts with membrane operation because aeration is commonly used for scouring and mixing; thus, DO setpoints should be optimized to balance granule stability, antibiotic biodegradation, and filtration stability.

#### 5.1.2. Hydraulic Shear Force

Hydraulic shear force has a significant impact on the granulation process, mainly affecting the size, morphology, polysaccharide production, cell surface hydrophobicity, and organic matter content of granular sludge. Granules formed under higher shear forces are more compact, dense, smooth, and have smaller particle sizes. Shear force determines the surface hydrophobicity of cells by promoting the release of polysaccharides from microbial cells, which in turn affects the formation and stability of granular sludge [54]. Furthermore, fluid shear force also influences the physiological activities of sludge. When hydraulic shear force is high, the energy produced by metabolism is mainly used for physiological reactions to produce polysaccharides rather than for increasing sludge quantity. Polysaccharides promote cell aggregation and adsorption, playing a crucial role in ensuring the structural integrity of granular sludge [109,110]. Increasing hydraulic shear elevates the EPS polysaccharide-to-protein ratio, mitigates bulking, and strengthens granular stability in SBRs [108].

Hydraulic shear force also has a significant impact on the structure and stability of granular sludge. Long-term high shear force can maintain high levels of DO, which can easily disrupt the granular structure. Appropriate hydraulic shear force aids granulation and maintains structural stability, enhancing settlement performance and biological activity. During antibiotic treatment, appropriately increasing hydraulic shear force can promote interactions between microorganisms, improving antibiotic degradation efficiency [111]. However, excessively high hydraulic shear force can lead to the breakage of granules, affecting their stability and treatment efficiency. In AGMBRs, shear is also a key lever for membrane scouring; therefore, shear management must consider a dual objective: suppressing membrane fouling without over-fragmenting granules into fines that can exacerbate filtration resistance.

#### 5.1.3. Sedimentation Time

Sedimentation time influences the granulation process, settlement performance, cell surface hydrophobicity, extracellular polysaccharide production, microbial activity and community, and cation accumulation. In SBRs, the cultivation of AGS mostly adopts the selective pressure method, which involves gradually shortening the sludge sedimentation time. When the sedimentation time is shortened rapidly, the selection pressure in the system is high, which can promote rapid and stable granulation. However, when the selection pressure is too high, the sludge concentration decreases sharply, which can also lead to system failure [112]. Therefore, the reasonable setting of sedimentation time is crucial for the formation of AGS. Short sedimentation times contribute to granule formation by promoting the accumulation of polyhydroxyalkanoates (PHA) within symbiotic organisms. During the feast phase, organic matter is stored in microbial cells in the form of PHA, which is then used for biomass growth during the famine phase [113,114].

For AGMBRs, this parameter should be interpreted carefully: because membrane separation can retain suspended biomass, classical settling-time selection may be weakened relative to conventional SBR granulation. As a result, AGMBRs often rely more on hydrodynamic shear, feast–famine regimes, and controlled biomass wasting to maintain a granular-dominant community. Thus, while sedimentation time remains mechanistically informative for granulation theory, its operational role may shift from being the primary selection tool to a secondary control interacting with membrane-retention-driven biomass management.

#### 5.1.4. Reactor Type

Reactor configuration is an important factor affecting the formation and stability of granular sludge. Cylindrical reactors using upflow aeration to provide shear force are conducive to granule formation. Additionally, SBRs are widely used in granule cultivation due to their flexible operation mode and high sludge concentration. Compared to sequential batch reactors (SBRs), continuous flow reactors (CFRs) exhibit better performance in terms of long-term stability of granular sludge. CFR systems achieve successful long-term granular stability through different inoculation methods, with the use of mature granular sludge pre-cultured in traditional SBR systems considered the best approach [115,116]. In CFRs subjected to elevated hydraulic shear, halophilic granular sludge (~420 μm) can be sustained alongside abundant EPS enriched in protein (81% of EPS) [117]. Up-flow sludge beds with carrier media enable treatment of high-nitrite wastewater starting from flocculent sludge [118]. A continuous-flow reactor with a two-zone sedimentation tank can cultivate granular sludge with ~105 μm particles and 26 mL/g SVI [119].

In the AGMBR context, reactor type further encompasses the membrane module configuration (e.g., submerged vs. side-stream, and associated hydrodynamics), which governs shear distribution, biomass–membrane contact frequency, and the accumulation of soluble/colloidal foulants near the membrane surface. Therefore, selecting and designing reactor types for SMX-containing wastewater should consider not only the ability to cultivate and sustain granules but also the capacity to maintain stable filtration performance under antibiotic-driven shifts in EPS and particle properties.

### 5.2. Water Quality Factors

In AGMBRs treating SMX-laden wastewater, influent water quality governs not only microbial growth and antibiotic biotransformation within granules but also the production of EPS/SMP and fine particles that can influence membrane-filtration stability. Accordingly, the following water quality factors should be interpreted through a dual lens: biodegradation capacity and process stability under membrane coupling.

#### 5.2.1. Organic Loading Rate

Organic matter concentration is a key factor affecting the growth and performance of granular sludge. Organic matter serves as the carbon source and energy source for microorganisms, and the organic loading rate (OLR) directly affects the growth rate and metabolic activity of microorganisms in sludge. At appropriate organic matter concentrations, microorganisms can rapidly reproduce and form stable granular sludge [120]. However, excessively high organic matter concentrations can lead to excessive sludge loading, affecting sludge settlement performance and the granulation process. High OLR can promote increased microbial activity and average particle diameter of granules [121]. Granular sludge can tolerate OLRs ranging from 2.5 to 15 kg COD/(m^3^·d). Excessively low or rapidly fluctuating OLRs can result in loose and porous granule structures, affecting system stability [122]. Alternating OLR (3.6–14.4 kg COD m^−3^ d^−1^) accelerates granulation (235 μm at day 17 vs. 179 μm at constant 7.2) and enriches tightly bound EPS protein [123]. Under alternating OLR conditions ranging from 3.6 to 14.4 kg COD m^−3^ d^−1^, granulation was markedly accelerated. Consequently, after 17 days, the average particle size reached 234.6 microns. In contrast, when the OLR was maintained at a constant level of 7.2 kg COD per cubic meter per day, the average particle size was only 179.2 microns. Furthermore, even after transitioning from alternating to constant OLR, the particle size continued to increase. The study revealed that alternating the OLR significantly boosted the content of EPS, particularly the protein (PN) found in tightly bound EPS (TB-EPS) [123].

In antibiotic wastewater treatment, an appropriate OLR ensures that microorganisms obtain sufficient substrate for growth and reproduction, thereby effectively degrading antibiotics [124]. Modulating OLR compacts fluffy particles into denser spheres and improves stability; mixed-biodegradability influent suppresses filamentous growth and prevents bulking [108]. In AGMBRs, this balance is particularly important because excessively high OLR can increase soluble/colloidal organics and EPS, potentially elevating fouling propensity, whereas excessively low OLR can limit microbial activity and slow antibiotic biodegradation. Therefore, reasonable regulation of OLR is crucial for sustaining both SMX removal efficiency and long-term filtration stability.

#### 5.2.2. Ammonia Nitrogen Concentration

Ammonia nitrogen concentration also has a significant impact on the formation and performance of granular sludge. Increasing the ammonia nitrogen concentration in influent water can promote granule formation because the oxidation process of ammonia nitrogen generates energy, which is beneficial for microbial growth and metabolism [125]. Additionally, ammonia nitrogen oxidation produces nitrate, providing an electron acceptor for the denitrification process, which is conducive to achieving simultaneous nitrification and denitrification [126]. Ammonia nitrogen concentration is crucial for the nitrification performance of granular sludge. Under low C/N ratios, granular sludge exhibits excellent nitrification performance, with NH_4_^+^-N removal efficiency close to 100% [127].

For AGMBRs, stable nitrification/denitrification within granules can support overall reactor function under SMX stress by maintaining redox stratification and functional guilds. Meanwhile, shifts in nitrogen conversion can indirectly influence EPS production and microbial community dynamics, which may alter the characteristics of foulants transported to the membrane surface.

#### 5.2.3. C/N Ratio

The C/N ratio is an important water quality parameter affecting the efficiency of granular sludge in degrading antibiotics. An appropriate C/N ratio ensures that microorganisms obtain sufficient carbon sources for growth and reproduction while meeting the demands of nitrogen metabolism. Under high C/N ratios, sludge granulation is slow, and settlement performance is poor. Low C/N ratios favor the accumulation of microorganisms within the reactor, but excessively low C/N ratios can lead to increased particle size, loose structure, and disintegration, with excessive proliferation of filamentous microorganisms, which is detrimental to system stability [128,129]. Therefore, a reasonable C/N ratio is crucial for the formation and performance of granular sludge. At a C/N of 4:1, granular sludge exhibits strong granulation and nutrient removal; more broadly, tuning the C/N ratio optimizes treatment of nitrogen- and phosphorus-containing wastewater [130]. Across SBRs run at C/N 6–10, a C/N of 8 produced stable, well-settling granular sludge with high pollutant-removal efficiency and rich microbial diversity [127].

In antibiotic wastewater treatment, excessively low C/N ratios may result in microorganisms lacking sufficient carbon sources, limiting their growth and metabolic activities, thereby reducing antibiotic degradation efficiency. Conversely, excessively high C/N ratios may lead to insufficient nitrogen sources, affecting the nitrogen removal capacity of microorganisms [131]. Optimizing the C/N ratio is key to improving antibiotic degradation efficiency. In granular sludge reactors, lowering the influent C/N markedly elevates roxithromycin and sulfamethoxazole removal to 95.2% and 92.9%, respectively [132]. The removal of ROX was found to be predominantly influenced by the adsorption capabilities of the granular sludge, whereas the removal of SMZ was primarily driven by biodegradation processes. Analysis of EPS revealed an enrichment of humic acid-like substances under low C/N conditions, which correlated with the dynamic shifts in the microbial community. In reactors subjected to high nitrogen loading rates, microorganisms such as Thauera spp. and Xanthomonadaceae were significantly enriched and played crucial roles in the degradation of refractory organics [133]. Overall, the study demonstrated that the granular sludge process can enhance drug removal performance by adjusting the microbial community under low C/N influent conditions.

For AGMBRs, C/N-driven shifts in EPS composition and microbial structure are additionally relevant to membrane operation because they can change the soluble/colloidal foulant spectrum. Thus, C/N optimization should target a window that simultaneously sustains SMX biodegradation pathways and avoids excessive production of fouling precursors.

#### 5.2.4. pH Value

The pH value is one of the key factors affecting microbial activity and antibiotic degradation efficiency. pH directly modulates the ionization state of functional enzymes, cell membrane permeability, and microbial community structure, as most hydrolytic/degrading bacteria involved in SMX biodegradation exhibit optimal activity in a near-neutral range. Different microorganisms have varying adaptability to pH values. A suitable pH range helps maintain microbial activity and promote antibiotic degradation [130]. For oxytetracycline biosorption on granular sludge, near-neutral pH (~6–8) yields optimal performance [134]. For SMX in AGMBRs, pH determines the speciation of SMX (molecular/ionic form): neutral SMX is more easily adsorbed by EPS/hydrophobic granular surface, while ionic SMX has higher water solubility and weaker biosorption, which directly affects the removal pathway partitioning. In granular sludge systems, fluctuations in pH may affect sludge structure and stability by altering EPS surface charge and inter-particle electrostatic repulsion; over-acidic/alkaline conditions can disrupt granular compactness and cause particle fragmentation, thereby influencing antibiotic degradation efficiency. Therefore, reasonable control of pH is of great significance for achieving efficient antibiotic degradation.

In AGMBRs, pH also regulates antibiotic speciation and electrostatic interactions between EPS/colloids and the membrane surface, and acidic/alkaline conditions enhance the adhesion of charged foulants to the membrane, increasing irreversible fouling and reducing cleaning efficiency, which can influence filtration resistance and cleaning behavior, making pH management a shared lever for both biodegradation and fouling control. The optimal pH window for AGMBRs treating SMX is 6.5–8.0, which balances microbial degradation, SMX adsorption, and membrane fouling mitigation.

#### 5.2.5. Temperature

Temperature is an important factor affecting microbial growth rate and metabolic activity. Temperature governs the kinetic rate of enzymatic reactions involved in SMX biodegradation; a 10 °C rise within the suitable range typically increases microbial metabolic rate by 1.5–2.0 times (Q10 effect), directly accelerating SMX mineralization. Within an appropriate temperature range, microbial metabolic activity increases, enhancing antibiotic degradation efficiency [26,135]. Temperature shapes start-up dynamics: medium temperatures (20–30 °C) speed initialization, yet granulation can still proceed at low temperature [136]. Relatively low temperatures (10–15 °C) help mitigate bulking and enhance granular sludge stability in SBRs by reducing microbial growth rate and filamentous bacteria proliferation while improving granular compactness and settleability [108]. However, excessively high (>35 °C) or low (<10 °C) temperatures can adversely affect microorganisms by inactivating key degrading enzymes, disrupting cell metabolism, and reshaping microbial community structure, reducing their activity and thereby affecting antibiotic degradation effectiveness. Therefore, in practical applications, the temperature should be reasonably regulated according to microbial adaptability to achieve optimal antibiotic degradation efficiency.

For AGMBRs, temperature impacts not only biodegradation kinetics but also sludge rheology and EPS/SMP production. Higher temperatures promote EPS/SMP secretion and increase colloidal particle mobility, accelerating cake layer formation and TMP rise; lower temperatures reduce foulant mobility but slow down SMX biodegradation, which can translate into different fouling rates and TMP trajectories. Hence, temperature effects should be interpreted jointly in terms of SMX removal efficiency and filtration stability under long-term operation. The optimal temperature range for AGMBRs treating SMX is 20–30 °C, which achieves a trade-off between high biodegradation efficiency and low membrane fouling propensity.

#### 5.2.6. Effects of Real Wastewater Matrices and Co-Contaminants

Real wastewater matrices can modify SMX attenuation in AGMBRs by changing the competitive partitioning of SMX into EPS/granules versus dissolved organic matter, the availability of labile co-substrates that enable cometabolism, and the formation and composition of membrane foulants (EPS/SMP and fine colloids). Evidence directly relevant to SMX in granular sludge-seeded MBR operation is available from sewage containing multiple PPCPs, where SMX removal reached 77.83% and improved with longer SRT/HRT, suggesting that high biomass retention and prolonged contact time can partially compensate for matrix complexity [137].

Different water types may further shift SMX removal and stability. For instance, high-salinity antibiotic-manufacturing wastewater has been shown to support aerobic granulation within an MBR when using specialized inocula, implying that salinity, industrial organics, and particulate nuclei can reshape granule properties and, consequently, antibiotic partitioning and fouling propensity [138]. These observations support the need to interpret SMX removal windows in AGMBRs as matrix-dependent rather than universal.

Co-contaminants can affect SMX removal both biologically and physicochemically. Co-occurring antibiotics may impose combined inhibitory or selective pressures that restructure microbial communities and granule stability, thereby altering SMX biodegradation capacity [139,140]. Moreover, co-selectors frequently found in real wastewaters (e.g., microplastics) can amplify SMX toxicity to granules and enrich antibiotic resistance genes, implying that SMX performance should be evaluated together with co-selectors in risk-aware AGMBR assessments [141]. Overall, real-wastewater validation should report not only SMX removal but also matrix composition (salinity, COD fractions, co-antibiotic spectrum) and concurrent shifts in EPS/SMP and particle-size distributions to enable transferable operational guidance across water types.

### 5.3. Key Components and Activity in Granular Sludge for AGMBRs

In AGMBRs treating SMX-containing wastewater, the biological core remains granular sludge, but its functionality must be evaluated together with membrane-coupled constraints. In practice, the microbial community structure and EPS dynamics determine not only SMX biotransformation and resistance to antibiotic stress, but also the generation of soluble/colloidal foulants that influence membrane filtration stability. Therefore, “key components and activity” in AGMBRs should be framed as an integrated bio–physicochemical basis for simultaneously achieving high SMX removal and stable long-term operation.

#### 5.3.1. Microbial Species

The formation of granular sludge is the result of microbial competition, elimination, and synergistic effects. The species of microorganisms undoubtedly have a significant impact on the formation and stability of granular sludge. Granular sludge contains a variety of heterotrophic and autotrophic bacteria, such as ammonia-oxidizing bacteria, nitrite-oxidizing bacteria, denitrifying bacteria, and others. These microorganisms aggregate in layers within the granules, forming a unique layered structure conducive to simultaneous nitrification, denitrification, and organic matter removal. Different microbial species play crucial roles in the formation and stability of granular sludge [142]. Nitrospira and Gammaproteobacteria dominate the granular biomass formed in CFRs, while Adhaeribacter is predominant in SBRs [143].

The microbial diversity and structure within granular sludge are controlled by factors such as organics, pH, temperature, and the stage of granulation, which are difficult to control and lead to significant variations in granule properties. Under antibiotic pressure, microorganisms that tolerate and/or degrade antibiotics can become dominant, with other microbial populations synergistically assisting and guiding the degradation process [81]. Therefore, for AGMBRs targeting SMX, domesticating granular sludge with high-density populations of antibiotic-degrading microorganisms—and maintaining their functional stability under membrane-coupled operating conditions—is key to achieving efficient antibiotic removal.

**Figure 7:**
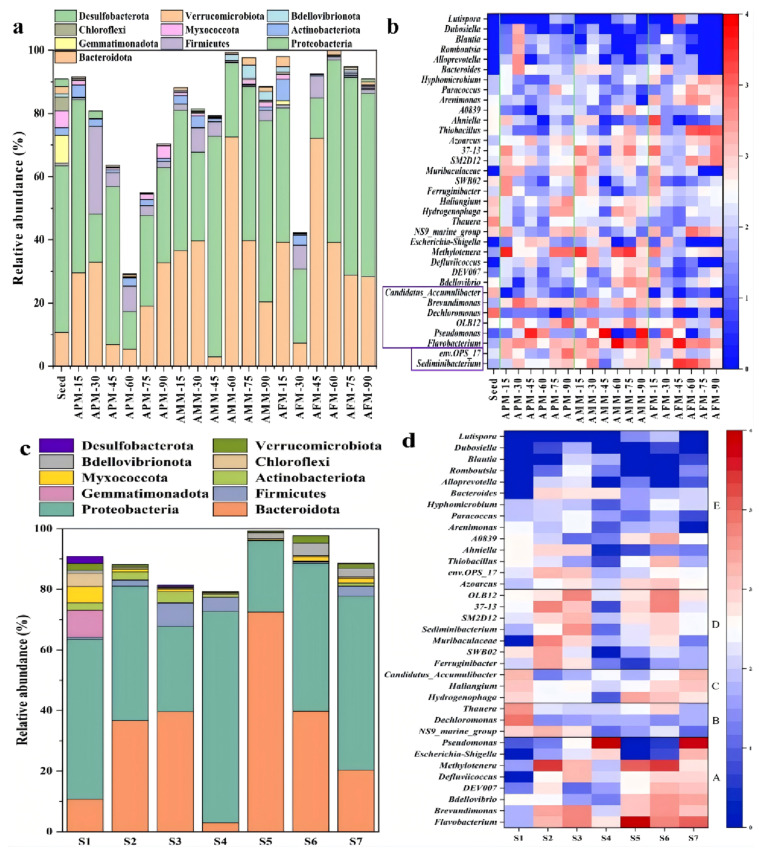
Diversity and composition of microbial communities in AGS: (**a**,**c**) Shifts in bacterial community composition at phylum level. (**b**,**d**) Heat map analysis of bacterial community composition at genus level [131] (community shifts are physically regulated by granular physical structure (porosity, compactness) and oxygen diffusion resistance across stratified micro-niches).

Across feeding modes, *Proteobacteria* and *Bacteroidota* dominate mature granules; physical constraints including granular size, pore structure and oxygen diffusion resistance (physical mass-transfer limits) favor the enrichment of *Firmicutes* in the anaerobic inner layer, and shifts among Dechloromonas, Thauera, and Candidatus Accumulibacter track granule stability and phosphorus removal (Figure 7a,b) [144]. During granulation, communities restructure—after 90 days *Proteobacteria* and *Bacteroidota* can reach 57% and ~20%, while Flavobacteriia falls to ~0.03%; denitrifiers such as Methylobacterium and Enterobacter–Shigella may replace Tetrasphaera and Dechloromonas (Figure 7c,d) [83]. This community succession is physically regulated by the evolution of granular physical structure (e.g., porosity, compactness) and hydraulic shear-induced physical screening. In an AGMBR setting, such succession patterns can be further shaped by membrane-enabled biomass retention and by filtration-linked stresses (e.g., accumulation of soluble microbial products), making community steering essential for balancing SMX degradation capacity with operational stability.

#### 5.3.2. The Role of EPS

EPS plays a significant role in the formation and stability of granular sludge. EPS, mainly composed of polysaccharides, proteins, and nucleic acids, exhibits adhesive and gel-forming properties that promote the aggregation and adhesion of microbial cells [145]. Serving as a “binder,” EPS facilitates the clustering of microorganisms. The synthesis of EPS is a vital component of granule formation, promoting biofilm formation among microorganisms like “glue.” Additionally, EPS acts as a protective layer for microorganisms, shielding them from external environmental pressures and adverse effects. EPS protects individual microbial cells from shock loads and toxic compounds [146]. Rising EPS correlates negatively with SVI and positively with granulation; the EPS PN/PS ratio can increase (1.42 to 4.17), while extracellular phosphorus rises then plateaus at 20%, consistent with EPS-mediated P removal [67].

In AGMBRs, EPS has a particularly important dual role: it strengthens granule cohesion and creates microenvironments that can enhance SMX partitioning and biodegradation, yet EPS (and associated soluble microbial products and colloids) is also a major contributor to membrane fouling. Thus, maintaining a high level of biological activity in granular sludge is necessary for SMX removal, but it must be achieved while managing EPS quantity and composition to avoid a shift toward filtration instability.

**Figure 8:**
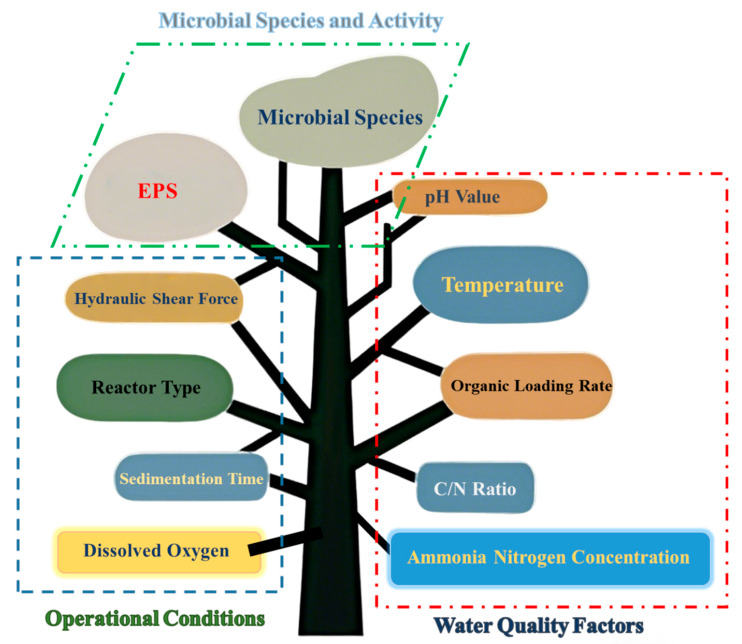
Key factors influencing the efficiency of aerobic granular sludge in degrading antibiotics.

Overall, antibiotic degradation in granular sludge is constrained by multiple interacting factors. By optimizing physical and chemical conditions such as dissolved oxygen concentration, organic loading rate, and hydraulic shear force, as well as water quality factors like C/N ratio, pH, and temperature, the antibiotic degradation efficiency of granular sludge can be significantly enhanced. Importantly, within AGMBRs these optimizations should be evaluated together with membrane-operation outcomes (e.g., EPS/SMP accumulation and filtration resistance) to ensure long-term stability. The key factors influencing the efficiency of granular sludge in degrading antibiotics are summarized in Figure 8. Future research should further explore the interaction mechanisms among these factors to provide theoretical support for developing more efficient and stable AGMBR technologies for SMX-contaminated wastewater.

### 5.4. Strategies to Enhance the Efficiency of AGS in Treating Antibiotic Wastewater

In the field of antibiotic wastewater treatment, AGMBRs have attracted increasing attention because they integrate the high-rate organic removal and nitrogen/phosphorus conversion capacity of granular sludge with membrane-based solid–liquid separation, thereby improving effluent quality and biomass retention under stress conditions. Long et al. discovered through experiments that in the final AGS formed under different feeding modes, *Proteobacteria* and *Bacteroidota* are the dominant phyla in all sludge samples, playing a key role in pollutant removal and ammonium nitrogen removal, respectively. As shown in Figure 7a,b, compared with seed sludge, the relative abundance of *Firmicutes* increased in all reactors, which is related to the limitation of oxygen transfer as particle diameters increase, promoting the growth of *Firmicutes*. However, under the impact of antibiotics such as sulfamethoxazole, improving the treatment efficiency and long-term operational stability of AGMBRs remains an urgent issue. This section summarizes strategies to enhance AGMBR performance from three aspects: (i) optimizing granular sludge cultivation conditions and reactor/membrane design, (ii) enhancing microbial activity and tolerance for sustained antibiotic degradation, and (iii) reducing treatment costs and improving economic viability.

#### 5.4.1. Optimizing Granular Sludge Cultivation Conditions and Reactor (Membrane) Design

Optimizing granular sludge cultivation conditions is the foundation for improving the treatment efficiency of AGMBRs. For inoculation and acclimation, mature aerobic granular sludge with high structural compactness and microbial diversity is selected as seed sludge, and gradient domestication under stepwise increasing SMX concentration is adopted to enhance the sludge’s tolerance and degradation potential toward antibiotics. Firstly, appropriate carbon and nitrogen sources should be selected to maintain the metabolic activity of microorganisms in the sludge. For antibiotic wastewater, easily degradable organics can be considered as co-substrates to improve the sludge’s degradation capacity for antibiotics [147]. A moderate C/N ratio (6–8) is regulated to promote the enrichment of functional degrading bacteria and reduce excessive EPS secretion that causes membrane fouling. Secondly, suitable temperatures, pH values, and dissolved oxygen concentrations should be controlled to create an environment conducive to microbial growth and reproduction [148]. Meanwhile, hydraulic shear force and settling time are precisely adjusted to enhance granule compaction, avoid particle fragmentation, and reduce the release of fine colloidal foulants.

In terms of reactor and system design, full consideration should be given to the granulation process and the filtration requirements introduced by membrane coupling [149,150]. For membrane module selection, submerged PVDF hollow-fiber membranes are preferred due to their high mechanical strength, good hydrophilicity, and low fouling propensity; membrane pore size is controlled at 0.1–0.4 μm to balance solid–liquid separation efficiency and filtration resistance. By adjusting the hydraulic retention time and aeration intensity, the formation and stability of granules can be promoted [151]. Aeration-induced shear is optimized to simultaneously achieve granule surface scouring, mass transfer enhancement, and membrane surface foulant stripping. Additionally, integrating granular sludge with membrane-based solid–liquid separation (i.e., membrane bioreactors) can further enhance removal efficiency and operational stability by strengthening biomass retention and mitigating biomass washout [152]. The decoupled control of SRT/HRT enabled by membrane retention is fully utilized to maintain high biomass concentration and prolong the contact time between microorganisms and SMX, thereby improving degradation efficiency. These reactor designs not only facilitate the retention and growth of sludge but also help prevent sludge loss and bulking [152]. The influence of reactor types on the formation and stability of AGS has been discussed in detail. Table 1 summarizes the specific parameters used for cultivating AGS in different membrane reactors.

The addition of fillers can further increase the enrichment density of granular sludge, exerting a shock-resistant effect on the degradation of antibiotics such as SMX in antibiotic wastewater. Complete Mixed Activated Sludge (CMAS), alternatively known as fully mixed activated sludge, stands as a highly efficient, cost-effective, and straightforward wastewater treatment process. It harnesses the power of microorganisms present in the activated sludge to degrade and eliminate organic pollutants from wastewater, achieving remarkable degradation rates through the thorough mixing of wastewater and activated sludge within the reactor. The CMAS system boasts numerous advantages, including robust adaptability, user-friendly operation, and reduced costs, making it an ideal choice for treating a wide range of industrial wastewater and municipal sewage. It particularly shines in the treatment of wastewater with high organic loads. Furthermore, the application of CMAS technology for the biodegradation of antibiotic wastewater using AGS presents a viable and promising option. Detailed operational parameters for this process are outlined in Supplementary Materials Table S1.

From an AGMBR viewpoint, these operational and design levers should be optimized not only for granule formation and SMX biotransformation but also for maintaining sustainable filtration (e.g., controlling the release of soluble/colloidal foulants that accelerate membrane fouling), thereby ensuring that gains in biodegradation are not offset by deteriorating membrane performance.

#### 5.4.2. Enhancing Microbial Activity and Tolerance for Antibiotic Degradation

Enhancing sludge microbial activity and tolerance is crucial for improving the efficiency of AGMBRs in treating antibiotic wastewater. On the one hand, microorganisms in the sludge can be domesticated to gradually adapt to the presence of antibiotics, thereby improving their degradation capacity [158]. This can be achieved by gradually increasing the concentration or variety of antibiotics to stimulate microorganisms to produce more degradation enzymes and adaptive mechanisms. On the other hand, microorganisms or genetically engineered bacteria with high-efficiency antibiotic degradation capabilities can be introduced to enhance the degradation capacity of the sludge [159]. Furthermore, by adding bioaugmentation agents such as activated carbon and zeolite, more attachment sites can be provided to promote microbial growth and reproduction, while also improving the sludge’s adsorption and degradation capacity for antibiotics [160].

In AGMBRs, these strategies can be particularly effective because membrane retention can help sustain slow-growing but functionally important degraders, while carrier/bioaugmentation approaches may simultaneously improve pollutant partitioning and stabilize community structure under SMX stress.

#### 5.4.3. Reducing Treatment Costs and Improving Economic Viability

While improving the efficiency of AGMBRs in treating antibiotic wastewater, reducing treatment costs and improving economic viability are also crucial. Firstly, energy and reagent consumption can be reduced by optimizing operational parameters and process conditions. For example, energy consumption can be lowered by precisely controlling aeration rates and reflux ratios; reagent consumption can be minimized by selecting appropriate chemical agents and dosages [161]. Secondly, the resource utilization of sludge can be explored, such as converting organics in the sludge into bioenergy or fertilizer, to realize waste resource utilization [162]. Hydrothermal carbonization (160–240 °C) retains >80% of phosphorus in bioavailable forms within hydrochar, with <5% released to the liquid, supporting resource recovery from AGS [163]. Additionally, cost-effective wastewater treatment technologies and equipment, such as high-efficiency and energy-saving aeration equipment and automation control systems, can be considered to reduce treatment costs [164].

**Figure 9:**
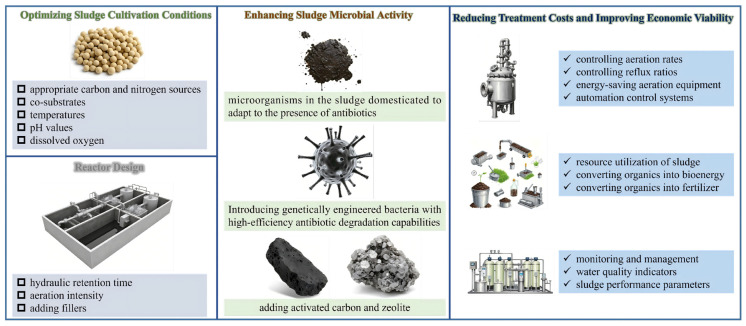
Strategies to enhance the efficiency of AGS.

Finally, monitoring and management during the wastewater treatment process should be strengthened to ensure the stability and reliability of treatment effects [165]. By real-time monitoring of water quality indicators and sludge performance parameters, operational parameters and process conditions can be adjusted in a timely manner to ensure the efficiency and economic viability of the wastewater treatment process. Strategies to enhance the efficiency of granular sludge systems for treating antibiotic wastewater are shown in Figure 9.

Overall, strategies to enhance antibiotic-wastewater treatment efficiency in AGMBRs include optimizing granular sludge cultivation conditions and reactor/membrane design, enhancing microbial activity and tolerance, and reducing treatment costs while improving economic viability. The implementation of these strategies will contribute to the broader application and development of AGMBR-based technologies for antibiotic-contaminated wastewater treatment.

## 6. Foresight

At present, AGMBRs still face several bottlenecks in treating antibiotic-containing wastewater. Real influent typically comprises mixtures of antibiotics together with co-selectors such as metals, disinfection by-products, and microplastics, producing pronounced non-linear and non-additive impacts on system performance and ARG dynamics. Although evidence for SMX removal is accumulating, complete reaction pathways, key enzyme systems, and the persistence, toxicity, and re-antibiotic activity of transformation products (TPs) remain insufficiently resolved. Most studies emphasize ARG abundance, with far fewer quantifying mobile genetic elements (MGEs) and the risks of horizontal gene transfer (HGT) in parallel with treatment performance.

On the process side, work is still dominated by bench-scale/short-term studies; evidence for long-term stability and recovery under shocks of loading, dissolved oxygen (DO), salinity, and temperature is limited. In AGMBRs specifically, antibiotic stress can also amplify membrane-relevant instabilities by reshaping EPS/SMP release and particle-size distributions, yet quantitative coupling among EPS composition (PN/PS and humic-like fractions), granular mechanics/mass transfer, and outcomes in biodegradation, fouling trajectories, and ARGs still lacks standardized methodology. Meanwhile, actionable set points and dynamic control strategies for the main “levers” (DO, organic loading rate—OLR, shear, etc.) have not yet been consolidated for antibiotic-rich influent, and scattered techno-economic assessment (TEA) and life-cycle assessment (LCA) baselines constrain deployment.

To address these gaps, future research should first construct mechanism maps for SMX and antibiotic mixtures that link enzyme-level steps, cometabolic nodes, and transformation products, while explicitly considering how membrane-coupled biomass retention and filtration constraints reshape exposure time and stress responses. Targeted and untargeted high-resolution mass spectrometry combined with effect-directed analysis can establish traceable links between parent/TP mass balances and residual bioactivity or ecotoxicity. In parallel, ARG risk should be quantified as a process variable: monitor ARGs together with MGEs, HGT proxies, and the viable resistome in the effluent, and partition them by phase (liquid vs. granule; extracellular vs. intracellular DNA) while jointly tracking treatment performance and filtration stability.

Next, adopt a structure–function engineering perspective tailored to AGMBRs: use shear, selection pressure, and co-substrates to deliberately tune EPS (PN/PS ratio and humic-like components), granule size, and porosity, thereby optimizing diffusion–reaction coupling while suppressing the generation of soluble/colloidal foulants that accelerate fouling. Design stress-test matrices across DO, OLR, C/N, shear, pH, and temperature to derive reusable control charts and standard operating windows that balance bulking control, high transformation rates, and stable filtration.

At the community and strain level, enrichment/domestication/engineering strategies can reinforce degrading guilds, while mineral additives (e.g., montmorillonite) should be evaluated for their ability to suppress ARG emergence. These interventions ought to be implemented with biosafety guardrails—containment and rollback plans—to ensure no increase in ARG mobility risk. To enhance robustness under complex influent conditions, explore how best to hybridize granular sludge with advanced oxidation, selective adsorption, or membrane processes so as to pre-activate or partition antibiotics and reduce the biological burden, emphasizing true synergy rather than simple additivity. Move beyond bench scale to multi-month pilots on real wastewaters from hospitals, pharmaceutical manufacturing, and livestock facilities, with standardized reporting of influent spectra, co-selectors, and shock scenarios; conduct TEA/LCA in parallel and include ARG risk reduction as part of the benefit metric set.

Finally, data and modeling infrastructure are essential. Release FAIR datasets with unified metadata (antibiotic panels, co-selectors, DO/OLR/C/N, EPS, ARG/MGE), and integrate multi-omics with reactive-transport modeling to develop digital twins that can predict performance and back-calculate control set points for new influents. Pursuing this roadmap can close the loop from molecular mechanisms → granular structure–function → controllable operating windows → pilot-scale techno-economic viability, directly confronting core challenges (mixtures and TPs, ARG mobility, robustness under shocks, and membrane-related stability) and providing verifiable, deployable scientific and engineering evidence for AGMBR treatment of antibiotic-laden wastewaters.

## 7. Conclusions

This review synthesizes advances and challenges for AGMBRs treating SMX-contaminated wastewater. (i) Regarding the impact of SMX, existing evidence shows that SMX can reshape microbial activity and community structure, perturb granule integrity, and consequently affect conventional pollutant removal; in AGMBRs these biological disturbances may further propagate to filtration stability by altering EPS/SMP release and particle-size distribution. (ii) Regarding the key operational and environmental factors, process levers such as DO, OLR, C/N, shear, pH, temperature, and reactor configuration jointly regulate diffusion–reaction conditions within granules and determine treatment outcomes; importantly, in AGMBRs they must be optimized in tandem with membrane operation to avoid trading improved SMX removal for accelerated fouling or unstable TMP/flux behavior. (iii) Regarding the mechanisms underpinning resilience and adaptability, AGMBR robustness arises from the stratified granule micro-niches, EPS-mediated partitioning/protection, metabolic versatility (biotransformation and cometabolism), and membrane-enabled biomass retention that sustains functional guilds under chronic exposure; strengthening this resilience calls for structure–function engineering of granules (size/porosity/EPS composition), community steering (enrichment/domestication/bioaugmentation), and synergistic hybridization with complementary units, together with integrated management of ARG risks (including MGEs/HGT) and long-term pilot validation. Addressing these priorities will help translate AGMBRs from laboratory demonstrations to scalable, reliable solutions for antibiotic-contaminated wastewater treatment.

## Figures and Tables

**Table 1:** Standardized parameters for cultivating AGS in different membrane-coupled reactors.

Process Configuration	Operation Mode	Wastewater Type	Granules Size/µm	HRT/h	Biomass Concentration/(g/L)	Target Pollutant Removal (%)	Ref.
UASB + side-stream membrane contactor	Continuous-flow anaerobic	Penicillin fermentation residue hydrothermal filtrate	2000–4000	120	-	COD: 75.0	[153]
EGSB-AnMBR	Two-stage continuous-flow	Synthetic swine wastewater with CIP (150 μg/L)	-	161	0.10–0.15	CIP: 98.6	[154]
MMBC (PVDF hollow fiber MBR)	Continuous-flow (light/dark)	Hospital wastewater with SMX (0–10 mg/L)	-	24	3.0	SMX: 41.07–50.15	[155]
Continuous-flow AGS-MBR	Continuous-flow	Synthetic wastewater with SMX (0–4 mg/L)	>450 (Dv50)	-	7.96–13.93	SMX: >95	[156]
MBGS-UF	Aerobic photobioreactor + UF	Municipal wastewater with SDZ (1/10 mg/L)	1700	8	8.82–10.13	SDZ: 94.1	[157]

## Data Availability

The original contributions presented in this study are included in the article/supplementary material. Further inquiries can be directed to the corresponding authors.

## Supplementary Materials

The following supporting information can be downloaded at: https://www.mdpi.com/article/10.3390/membranes16040139/s1, Table S1: Reported Effects of SMX on Effluent Quality and Process Performance in AGS Systems.
