# Supplementary material for: Advances and Challenges in Aerobic Granular Sludge Membrane Bioreactors for Treating Sulfamethoxazole in Wastewater
Source: Membranes (Basel). 2026 Apr 1;16(4):139. doi: 10.3390/membranes16040139 (PMC13118233; doi:10.3390/membranes16040139)
Supplement: Supplementary file 1 [file membranes-16-00139-s001.zip › membranes-4231324-Supplementary Materials.pdf]

Table S1 Reported Effects of SMX on Effluent Quality and Process Performance in AGS Systems

Scale & System	SMX Dose	Operating Conditions	COD/TOC Removal Change	NH_4^+-N & TN Removal Change	TP Removal Change	Effluent SS/Turbidity Change	Effluent SMX Residual	Ref.
Lab-scale AGS-SBR (R2) with control reactor (R1)	200 μg L^{-1} SMX (plus 200 μg L^{-1} TMP)	Aerobic granular sludge SBR; control vs antibiotic-fed reactor; AQDS (25 μM) added at later stage	Similar to control; no clear deterioration in organic removal	Similar overall C/N/P removal to control; with AQDS, NH_4^+-N and TN removal increased	Similar to control; AQDS caused little change in P removal	AGS structural integrity impaired by antibiotics	~80 μg L^{-1} without AQDS (based on ~60% SMX removal); ~10 μg L^{-1} with AQDS (based on ~95% removal)	[1]
Lab-scale GSBR (R1 with SMX start-up; R2 control start-up)	5 μg L^{-1}	Granular sequencing batch reactor; start-up comparison with and without trace SMX	Both systems maintained good removal of conventional pollutants	NR	NR	No direct effluent SS/turbidity data; granulation not inhibited, but granules in SMX-started reactor were smaller	~1.30 μg L^{-1} in SMX-started reactor and ~1.47 μg L^{-1} in control-started reactor after stabilization (estimated from ~73.93% and ~70.66% removal)	[2]
Lab-scale AGS reactor, continuous 240-day test	0.5–5 mg L^{-1}	Long-term continuous operation; stepwise SMX loading after mature AGS formation	NR in abstract/source excerpt	Strong resistance at 0.5 mg L^{-1}; at 1 and 5 mg L^{-1}, nitrification efficiency declined markedly	NR	No direct effluent SS/turbidity data; sludge settleability weakened and filamentous overgrowth occurred at 1–5 mg L^{-1}	Thoroughly removed after acclimation; exact effluent concentration not directly reported in abstract/source excerpt	[3]

Table S1 Reported Effects of SMX on Effluent Quality and Process Performance in AGS Systems

Scale & System	SMX Dose	Operating Conditions	COD/TOC Removal Change	NH_4^+-N & TN Removal Change	TP Removal Change	Effluent SS/Turbidity Change	Effluent SMX Residual	Ref.
Lab-scale FAGS-CFR (filamentous aerobic granular sludge, continuous-flow reactor)	2 μg L^{-1}	Long-term continuous-flow reactor treating SMX; filamentous AGS system	COD removal remained >85%	NH_4^+-N removal remained >80%; TN not directly reported	NR	No direct effluent SS/turbidity data; system remained stable during long-term operation	<0.4 μg L^{-1} (estimated from >80% SMX removal)	[4]

Notes:

NR = not reported directly in the abstract or accessible source excerpt.

For the “Effluent SMX residual” column, values were estimated from influent SMX concentration × (1 − removal efficiency) when only removal efficiencies were reported. The Zhang et al. study stated “thorough removal” of 0.5–5 mg L^{-1} SMX after acclimation, but the exact residual concentration was not given in the accessible abstract.

References

[1] Barros, A.R.M.; Argenta, T.S.; de Carvalho, C.A.; Oliveira, F.S.; Firmino, P.I.M.; dos Santos, A.B. Effects of the antibiotics trimethoprim (TMP) and sulfamethoxazole (SMX) on granulation, microbiology, and performance of aerobic granular sludge systems. Chemosphere 2021, 262, 127840. [Google Scholar] [CrossRef]

[2] Cui, D.; Wei, N.; Ling, N.; Zheng, G.; Sun, Y.; Chen, Z.; Zou, X.; Deng, H.; Li, W. Effects of sulfamethoxazole on aerobic sludge granulation process. J. Appl. Microbiol. 2022, 132, 1091–1103. [Google Scholar] [CrossRef]

[3] Zhang, B. et al. Biotransformation of sulfamethoxazole (SMX) by aerobic granular sludge: Removal performance, degradation mechanism and microbial response. Sci. Total Environ. 2023, 858, 159771. [Google Scholar] [CrossRef]

[4] Song, T.; Zhang, X.; Li, J.; Xie, W.; Dong, W.; Wang, H. Sulfamethoxazole impact on pollutant removal and microbial community of aerobic granular sludge with filamentous bacteria. Bioresour. Technol. 2023, 379, 128823. [Google Scholar] [CrossRef]
